# Relational Modelling for Automotive Cybersecurity: Structural Transition and Graph-Topology-Based CAN Intrusion Detection

**DOI:** 10.3390/s26102964

**Published:** 2026-05-08

**Authors:** Mohammad Khalaf Khreasat, Gabriel Villarrubia González

**Affiliations:** Expert Systems and Applications Laboratory (ESALab), Faculty of Science, University of Salamanca, 37008 Salamanca, Spain; gvg@usal.es

**Keywords:** Controller Area Network (CAN), intrusion-detection system, graph topology, structural transition features, cross-attack evaluation, automotive cybersecurity, machine learning, anomaly detection

## Abstract

A central open question in automotive intrusion detection is not merely whether relational representations of Controller Area Network (CAN) traffic improve performance, but *which aspects of CAN traffic structure transfer robustly across attacks and which do not transfer across vehicle platforms, and why.* To investigate this question systematically, we develop a lightweight intrusion-detection framework combining statistical traffic descriptors, structural identifier transition features, and graph topology representations extracted from sliding windows of CAN frames. Because CAN is a broadcast-only bus with no request–response mechanism, each ECU independently transmits its identifiers at fixed periodic rates; accordingly, the structural and graph-based features capture the *temporal scheduling regularity* of identifier broadcasts, not directed inter-ECU communication dependencies. Stress-testing the framework under cross-attack and cross-dataset transfer reveals a clear four-level hierarchy: (1) statistical features collapse under cross-attack transfer (ROC-AUC as low as 0.009), failing to generalise beyond the attack type seen during training; (2) structural transition features are the most robust form of representation, maintaining high cross-attack performance (ROC-AUC > 0.999) across all evaluated scenarios within the same vehicle platform; (3) graph topology features are scenario-dependent, achieving high robustness in DoS-trained scenarios but producing sub-random results in Fuzzy-trained scenarios, exposing a sensitivity to injection density profiles; and (4) the hybrid combination provides the strongest overall operational package, consistently across four classifiers. Cross-dataset transfer to the ROAD dataset reveals the precise boundary conditions of transferability: structural representations transfer only when an attack perturbs identifier transition regularity (correlated signal attacks, ROC-AUC = 0.81–0.83), while attacks that affect only payload semantics (speedometer) or exploit identifier–space novelty (fuzzing) lie outside the detection scope of transition-based features, regardless of the vehicle platform. A vehicle-specific calibration experiment further shows that the correlated-attack generalization gap can be closed with as little as 10% of target-vehicle normal traffic, whereas speedometer attacks remain structurally invisible by design. A key contribution of this work is therefore a transparent approach for identifying when relational CAN representations transfer and when they do not—a finding that is more scientifically valuable than a uniformly optimistic performance claim and which provides concrete guidance for practitioners designing cross-platform automotive IDS.

## 1. Introduction

Vehicles have become increasingly complex and sophisticated; each modern vehicle contains hundreds of electronic control units (ECUs), many of which play a role in the vehicle’s critical functions, such as engine management, braking, steering, driver assistance functions and infotainment [1]. Each ECU communicates with other devices in the vehicle through an in-vehicle network; the majority of these in-vehicle networks utilize the Controller Area Network (CAN) protocol [2]. While the CAN protocol is efficient and reliable, it lacks built-in security functions such as authentication and encryption [3]. Therefore, any device attached to the CAN bus can send messages [3]. Malicious actors have successfully exploited the lack of security within the CAN protocol to affect vehicle function by manipulating vehicle signals, disrupting communication between ECUs or injecting false information into vehicle systems, affecting how the vehicle operates [4].

As a response to the threat of malicious actors, numerous CAN-based intrusion-detection system (IDS) proposals have been developed [5]. Most existing IDS approaches use statistical traffic descriptions such as timing patterns of messages, identifier distributions and payload statistics [6]. However, the robustness of existing IDS approaches to attacks that vary in terms of their behavioural characteristics has yet to be determined [7].

Specifically, models trained on one type of attack may fail to identify attacks with varying behavioural characteristics due to the fact that statistical features typically only describe marginal traffic properties and not the temporal, sequential co-occurrence structure of CAN identifiers in the broadcast stream [8].

To remedy this issue, the focus of this paper will be structural representations of CAN broadcast ordering [9]. Rather than examining CAN messages individually, we will examine the temporal, sequential patterns formed by identifier broadcasts, i.e., the co-occurrence ordering produced by the deterministic periodic transmission schedules of ECUs [10]. Specifically, we will represent CAN traffic windows using statistical descriptors, structural transition features and graph topology metrics extracted from identifier transition graphs [9].

Our central hypothesis in this work is that the temporal broadcast ordering patterns exhibited by CAN traffic are much more stable when the attack vector changes than the marginal statistical properties [11]. Furthermore, since the structural and graph-based representation captures the temporal scheduling regularity of identifier broadcasts rather than attack-specific marginal statistics, it is expected to exhibit improved cross-attack generalisation compared to traditional statistical representations. Cross-dataset generalisation is expected to be attack-type-dependent, as it requires the structural disruption pattern to be consistent across vehicle platforms [12].

To test our hypothesis, we performed experiments utilizing the HCRL Car-Hacking dataset [13]. We utilized six machine learning classifiers: Logistic Regression, Support Vector Machine, Decision Tree, K-Nearest Neighbors, Random Forest, and Gradient Boosting. Notably, while most classifiers demonstrated consistent performance across feature representations, the Decision Tree classifier exhibited instability when evaluated with hybrid feature sets, achieving a mean ROC-AUC of 0.527 across cross-attack scenarios. This instability is attributed to the tendency of Decision Trees to overfit to specific feature interactions, particularly when the feature space combines heterogeneous statistical, structural, and graph-based descriptors. By testing multiple classifiers, we will be able to ascertain whether the robustness of our proposed method stems from the features alone or from the specific classifier used [14].

The experimental results indicate that statistical features may fail when transferred to cross-attack scenarios, resulting in almost random detection performance in some instances [15]. On the other hand, structural transition features exhibited consistently high detection performance across classifiers [16], while graph topology descriptors were informative but scenario-dependent. Our results illustrate the need for structural broadcast ordering modelling to develop robust automotive intrusion-detection systems [17].

Finally, the primary contributions of this paper are listed below:

1. We demonstrate the limitations of statistical CAN intrusion-detection methods under cross-attack evaluation scenarios.
2. We propose a lightweight feature extraction framework combining statistical, structural, and graph-based representations of the CAN broadcast stream structure.
3. We evaluate the robustness of the proposed approach across multiple machine learning classifiers.
4. We show that structural broadcast ordering modelling significantly improves detection robustness compared with purely statistical traffic descriptors.
5. We conduct a window size sensitivity analysis demonstrating that the proposed framework maintains stable detection performance across window sizes ranging from 50 to 500 CAN frames, validating the robustness of the approach to this hyperparameter choice.

## 2. Related Work

Several recent works have investigated the use of Deep Learning for CAN-based intrusion detection [17].

Recurrent Neural Networks (RNNs), specifically Long Short-Term Memory (LSTM) architectures, have been proposed to capture the temporal dependencies within CAN traffic sequences [18].

Convolutional Neural Networks (CNNs) have been utilized to develop spatial representations of patterns of CAN messages [19].

These Deep Learning approaches demonstrated promising detection results; however, they are typically limited by requirements for large amounts of labelled data and considerable computational resources [20]. Moreover, deep models may be trained to recognize dataset-specific patterns which are less likely to generalize across different vehicles and/or attack scenarios [21].

Other networked systems (i.e., power grid monitoring, communication networks (social network analysis) have employed graph-based anomaly-detection methods [22]. The graph-based approach models the interactions between entities as graphs and analyses topological properties to detect abnormal patterns [23].

Influenced by previous research, this work employs a directed graph approach to represent CAN identifier transitions and extracts topology features that describe the higher-order structural properties of the identifier broadcast sequence observed on the CAN bus [9].

### 2.1. Statistical CAN Intrusion Detection

Early CAN intrusion-detection systems were based primarily upon the statistical analysis of how traffic behaved [24]. A timing-based approach is possible because of the periodic nature of how electronic control units ECUs send data (messages) over CAN, which can be determined by their control loop execution rates and sensor sampling periods [10]. Therefore, an ECU sending data at times different than expected could indicate the presence of malicious actions (message injection or denial-of-service (DoS) attack) [10]. Statistical methods, in addition to identifying deviations from normal transmission timing, also identify abnormalities within the distribution of the identifier entropy and/or payload values to identify anomalies within traffic patterns [25].

Although statistical methods are very low cost to implement and embed into an ECU, they represent the first order characteristics of traffic and therefore have a common weakness: they analyse each message in isolation with no consideration of the relational dependency of the identifiers [26]. Therefore, if an attacker crafts a message that preserves all the statistical properties of valid messages but causes a disruption of the legitimate communication structure, then it will be difficult to detect via this type of method [27].

### 2.2. Machine-Learning-Based CAN IDS

Most studies have used supervised learning (Random Forest, Support Vector Machine, Logistic Regression) for intrusion detection in CAN networks; these are often trained with features that were manually created based on traffic statistics, payload, and identifiers [28]. Those models create decision boundaries between normal communications and malicious communications, and generally perform well when there are no other attacks or network configurations than those used during training [29].

One major flaw in the vast majority of machine learning-based studies of CAN IDS is their test methodology [30]. Typically, models are both trained and evaluated on the same attack type and vehicle platform [31]. As such, this testing environment is likely to be less representative of the actual deployment scenarios of IDSs in vehicles; it is possible that different types of attacks will occur, and the vehicle’s communications environment will also be very different [30]. Therefore, the ability of those models to generalize across attacks and datasets has not been extensively studied [32].

### 2.3. Deep Learning Approaches

Convolutional Neural Networks (CNNs), Recurrent Neural Networks (RNNs), and a subset of RNNs called Long Short-Term Memory (LSTM) networks have been used to capture temporal dependency from message sequence that is generated by CAN messages [33]. In particular, LSTMs provide an effective method for modelling sequential data and have demonstrated strong detection performance when compared to traditional CAN intrusion detection benchmarks [21].

Although Deep Learning models can be very powerful in representing complex patterns within data, there are two major practical challenges that need to be overcome before Deep Learning models can be used in automotive applications [34]. The first challenge is that most deep learning models require access to large amounts of labelled data to train on and also consume a lot of processing power [35]. These requirements make it difficult to implement Deep Learning models in many resource-constrained ECUs [17]. The second challenge for Deep Learning models is that they typically learn specific patterns that are contained within a given training dataset and do not generalize well to other types of communication [36]. The tendency of these models to overfit to the training distribution is thought to be one reason why the deep models tend to perform poorly when tested under cross-attacks or when transferred across different datasets [37].

### 2.4. Structural Modelling of CAN Traffic

Transition-based structural modelling methods examine the relationship among CAN IDSs as opposed to examining only marginal traffic statistics for each IDS. Due to the deterministic control logic and scheduled message transmission of ECUs in a vehicle, it is expected that CAN message sequences will have a stable temporal order determined by the fixed periodic transmission schedules of individual ECUs. Transition-based models capture this through representation of the sequential co-occurrence between successive identifier broadcasts. Under typical operational conditions, the transitions of identifiers can be very predictable due to the deterministic nature of the ECU communication logic. The presence of malicious messages from an attacker could cause abnormal transitions between identifiers to occur, even though all traffic statistics appear to be normal. Therefore, the use of transition-based structural features provides a detection mechanism that is complementary to and enhances traditional marginal statistical analysis.

### 2.5. Graph-Based Intrusion Detection

Graph-based anomaly-detection methods utilize entities of systems as nodes and interactions between those entities as edges. In a CAN network, the identifiers can be represented as nodes, while transitions between identifiers are directed edges which enable the analysis of the broadcast ordering structure using metrics of graph topology. Graph topology metrics capture structural properties of the identifier broadcast sequence such as node degree distributions, graph density, and entropy measures that extend beyond local pairwise transition observations.

There is increasing research interest in applying Graph Neural Networks (GNNs) to intrusion detection in complex networked systems. Although GNN-based models demonstrate significant representation capability, they often require large amounts of training data and computational resources, which may not be available in embedded automotive environments.

The approach proposed in this work focuses on lightweight descriptors of graph topology derived from identifier transition graphs. Instead of learning deep graph embeddings, the proposed framework extracts structural metrics that are interpretable such as graph density, statistics of node degrees, and transition entropy. By reducing computational complexity, the proposed framework captures the temporal scheduling regularity of identifier broadcasts, which improves robustness under cross-attack conditions. Cross-dataset performance is attack-type-dependent and limited, as detailed experimentally in Section 6. The framework provides a practical alternative to deep GNN architectures for real-time deployment in automotive applications.

## 3. Theoretical Motivation for Structural Modelling

Is there a pattern of structural stability in traffic that can be found when vehicles operate normally? The electronic control units of vehicles send periodic messages about their state based on predefined control loops and vehicle dynamics. Therefore, sequences of identifiers are formed into stable temporal broadcast ordering patterns determined by the fixed periodic transmission schedules assigned to each ECU at the time of designing. The purpose of this section is to describe why we expect that structural or graph-based representations of data will produce more accurate and robust anomaly detection than the application of marginal statistical methods for aggregating data.

### 3.1. Structural Transition Modelling

The advantage of structural transition modelling is that it captures the temporal co-occurrence ordering of successive identifiers in the broadcast stream. Rather than treating each identifier as an independent observation, structural transition models represent the sequential regularities produced when multiple ECUs transmit at their respective fixed periods, causing their broadcasts to interleave in a predictable pattern. This ordering is stable under normal operating conditions because ECU transmission periods are defined at design time and do not vary with vehicle state.

The structural transition entropy quantifies the ability to anticipate or predict the identifier sequences contained in a specified communication window. Normally, under normal vehicle operational conditions, each ECU broadcasts on its predefined fixed periodic schedule, thus generating predictable and consistent low-entropy identifier transition patterns (i.e., consistent temporal ordering of identifier broadcasts). Malicious injections into the CAN traffic can generate abnormal identifier transitions, regardless of whether the marginal statistical properties of the traffic have changed. Thus, elevated structural transition entropy generates a detection signal that supplements the detection signal provided by marginal statistical anomaly detection.

Structural transition representations have a greater capability to model higher-order temporal ordering regularities present in the CAN broadcast stream compared to the statistical representations used for anomaly detection. As long as an attacker does not alter the marginal frequency distribution of identifiers, the structural transition analysis has the potential to detect either new identifier orderings or previously unseen combinations of identifiers injected into the CAN traffic as a result of malicious activity. Since the structural transition analysis is less susceptible to evasion techniques that attempt to preserve statistical distributions, they provide an additional layer of robustness to such attacks.

### 3.2. Graph-Based Communication Topology

The graph representation is an extension of structural transition modelling to represent the global communication pattern throughout the time interval. Each unique CAN identifier has a corresponding node, while directed edges represent the transition between identifiers as they are observed.

**Figure 1:**
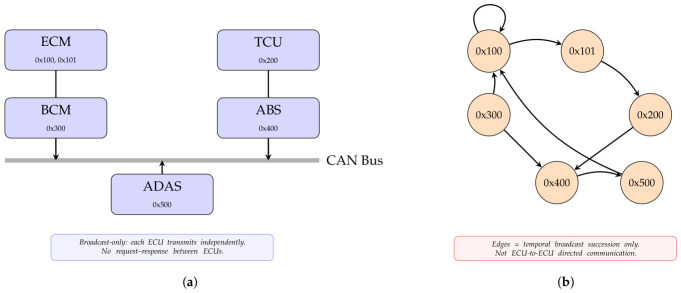
From CAN bus to identifier transition graph. (**a**) Typical in-vehicle CAN bus architecture: each ECU (ECM = Engine Control Module, TCU = Transmission Control Unit, BCM = Body Control Module, ABS = Anti-lock Braking System, ADAS = Advanced Driver-Assistance System) independently broadcasts messages using a fixed set of CAN identifiers on a deterministic periodic schedule. CAN communication is broadcast-only: transmissions are not conditioned on responses from other ECUs, and senders have no confirmation of reception. (**b**) Directed identifier transition graph extracted from a sliding window of CAN frames: nodes represent unique CAN identifiers, and directed edges capture the temporal, sequential co-occurrence of consecutive messages in the observed stream. Because each ECU transmits at a fixed rate, this co-occurrence structure is stable and predictable under normal conditions; edges therefore reflect *temporal scheduling regularity*, not directed communication dependencies between ECUs. Graph topology metrics derived from this representation quantify the structural regularity of the vehicle’s periodic broadcast schedule.

In CAN-based vehicular networks, each ECU broadcasts its messages using a fixed set of CAN identifiers assigned during vehicle design. This one-to-one (or one-to-few) mapping between ECUs and CAN identifiers is a fundamental architectural property of CAN networks, where message ownership is implicit in identifier assignment rather than explicitly encoded in the frame header [38]. It is important to note, however, that CAN communication is fundamentally broadcast in nature: each ECU independently transmits its assigned identifiers at fixed periodic rates determined by its control loop cycle time, without knowledge of whether any receiving node has processed the message [2]. There is no request–response mechanism, and transmissions are not conditioned on responses from other ECUs [2]. Consequently, identifier-level transitions in a CAN traffic trace do *not* encode directed communication dependencies between ECUs; rather, they reflect the deterministic temporal interleaving of independent periodic broadcasts. Because each ECU’s transmission period is fixed at design time [10], the sequential co-occurrence of identifiers in the stream is highly predictable and stable under normal operating conditions. It is this *temporal scheduling regularity*—not inter-ECU communication logic—that produces consistent identifier transition patterns. When an attacker injects messages carrying new or misused identifiers, the expected interleaving pattern is disrupted even if the marginal statistical properties of the traffic remain unchanged, as illustrated in Figure 1.

The graph topology metrics derived from this representation reflect the higher-order structural property of the CAN communication. The density of the graph represents the ratio of the number of observed identifier transition to the total number of possible transition for the given window. When the vehicle operates normally, the identifier broadcasts follow well-defined temporal interleaving patterns among the fixed set of periodic senders; therefore, the density of the transition graph will remain within a relatively narrow range. However, the introduction of malicious message injection may create new transition and/or modify the existing transition, thus breaking the regularity of the structural relationship.

The entropy of degree captures the variation in identifier connectivity and reflects the hierarchical broadcast frequency structure of the vehicle’s ECUs. Under normal operating conditions, some identifiers correspond to ECUs that transmit at high frequency or whose broadcasts frequently precede those of many other identifiers, generating a characteristic degree distribution. Malicious message injection disrupts the periodic interleaving pattern and introduces unexpected co-occurrence transitions between identifiers.

Collectively, the above-mentioned topological metrics provide a global view of the broadcast ordering structure that goes beyond the local temporal co-occurrence patterns captured by structural features alone. The proposed framework provides a complementary layer of broadcast stream analysis by capturing both the local temporal transition patterns and the global identifier co-occurrence topology, and improves the robustness of the detection.

Within a sliding window, nodes correspond to CAN identifiers; meanwhile, directed edges represent sequential transitions between identifiers. This representation captures the temporal broadcast ordering structure of the identifier transmissions observed in the CAN stream.

### 3.3. Stability Hypothesis

The primary hypothesis in this research is that structural transition patterns are expected to be more stable than marginal statistical properties under cross-attack transfer within the same vehicle platform; meanwhile, cross-dataset transfer is expected to remain attack-type-dependent, varying with whether an attack perturbs identifier transition regularity or only payload semantics.

This is based upon the deterministic characteristics of ECU communication. Because each sequence of identifiers is determined by the control logic defined for the vehicle during the design process, normal transition patterns should remain consistent with respect to the dependency on hardware-level characteristics that exist regardless of operating conditions or vehicle platform.

On the other hand, marginal statistical properties (e.g., timing between arrivals, frequency of identifiers) will be much more sensitive to variations in driving conditions, the speed of the vehicle, and the load of the communications. These variables can introduce shifts in distributions that may result in marginal features behaving differently on different datasets even when all vehicles are operating normally—this reduces their reliability as cross-dataset anomaly detectors.

Therefore, by focusing on the temporal broadcast ordering structure of the CAN stream instead of marginal statistics, the proposed framework attempts to identify a representation of normal vehicle broadcast scheduling regularity that is both more stable across attack types and more robust for cross-attack generalisation. Cross-dataset generalisation is attack-type-dependent, as detailed in Section 6.

The experimental results shown in Section 6 empirically validate and qualify the hypothesis outlined above.

## 4. Methodology

**Figure 2:**
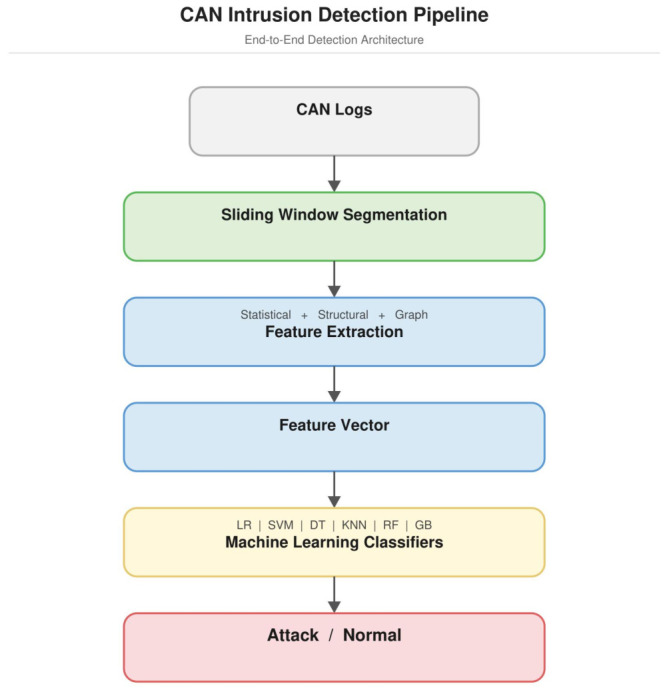
Overall architecture of the proposed CAN intrusion-detection framework.

The overall architecture of the proposed intrusion-detection framework is illustrated in Figure 2. The system processes raw CAN traffic logs and converts them into structured feature representations through several processing stages. First, CAN frames are segmented into sliding windows to capture short-term broadcast ordering patterns. For each window, statistical traffic descriptors, structural identifier transition features, and graph topology features are extracted.

These feature representations are then combined into a unified feature vector and used as input to the detection model. The model learns to distinguish between normal and disrupted broadcast ordering patterns based on these multi-level representations.

Raw CAN logs are segmented into sliding windows, from which statistical, structural transition, and graph topology features are extracted. The resulting feature vectors are evaluated using multiple machine learning classifiers.

In the experiments, multiple machine learning classifiers are evaluated to assess the robustness of the proposed feature representations. The evaluated classifiers include Logistic Regression (LR), Support Vector Machine (SVM), Decision Tree (DT), K-Nearest Neighbors (KNN), Random Forest (RF), and Gradient Boosting (GB).

### 4.1. Sliding Window Representation

Each window is labelled as malicious if it contains at least one attack frame, and as normal otherwise. This labelling strategy enables anomaly detection at the window level rather than at the individual frame level.

This labelling strategy makes it possible to label segments of communication as anomalous rather than labelling individual frames. When examining identifier sequences contained in each window, both the local properties of the communication (such as the diversity of identifiers over time, the variation in the timing of communication events, etc.) and the global properties of the communication (the structural relationships between identifiers) are examined. These graph representations reflect the temporal co-occurrence ordering of identifiers produced by the deterministic periodic broadcast schedules of the vehicle’s ECUs. By analysing the structural features of these graphs, such as transition entropy and node degree distribution, the proposed framework can identify anomalies that disrupt the normal scheduling regularity of periodic transmissions.

Based upon preliminary experiments, we determined that a window size of 200 frames was the optimal choice between detection stability and responsiveness. Windows of size 100 resulted in slightly lower Area Under the Receiver Operating Characteristic Curve (ROC-AUC) scores due to the limited structural context, whereas windows of size 300 did not result in a significant improvement in detection performance but introduced larger delays in processing. Therefore, a window size of 200 frames was used for all experiments.

It is important to note that this window-level labelling represents an inherent design trade-off. Relational features such as structural transitions and graph topology metrics are only meaningful when computed over a sequence of frames; they cannot be derived from a single frame in isolation. As a result, the framework operates as a window-level anomaly flag rather than a frame-level attribution mechanism. When a window of 200 frames contains even a single injected malicious frame, the resulting disruption to identifier transition sequences and graph topology is measurable and raises the detection signal, even though the remaining 199 frames may be legitimate. In practice, when a window is classified as malicious, the recommended response at the ECU or gateway level is to trigger an alert or initiate a deeper inspection mode rather than to discard all frames in the window. The precise action taken (logging, alerting, isolation) is a system-level policy decision that lies outside the scope of the IDS and must be defined by the vehicle manufacturer. Frame-level localization of the specific malicious frame within a flagged window is not supported by the current framework and is identified as a direction for future work (for example, via a lightweight, second-stage, frame-level classifier that operates only on windows already flagged as anomalous).

**Effect of attack frame density on apparent detection performance:** The window-level labelling strategy—labelling a window as malicious if it contains at least one attack frame—effectively redefines the detection task from identifying sustained anomalous patterns to detecting whether any attack frame is present within a window. This distinction is important for interpreting the reported results, because the apparent effectiveness of certain feature types can be amplified when the density of attack frames within positive windows is high.

In the HCRL Car-Hacking dataset, injection attacks (DoS, Fuzzy, Gear, RPM) operate by inserting frames at high and sustained rates, meaning that positive windows typically contain a large proportion of attack frames rather than isolated single injections. Under these conditions, the structural and statistical disruption per window is large, which tends to make detection easier regardless of the feature type. By contrast, in transfer scenarios (cross-attack or cross-dataset), the feature generalisation properties become the primary discriminator, and the high within-window attack density of the training distribution may not be representative of the test distribution.

To mitigate the risk of overestimating detection effectiveness, the reported ROC-AUC and PR-AUC metrics were computed across the full range of decision thresholds, which provides a threshold-independent assessment of discriminative power rather than accuracy at a single operating point. Furthermore, the cross-attack evaluation—in which models trained on one attack type are tested on a different attack type—provides the most stringent test of feature generalisation and is the primary basis for the paper’s conclusions.

### 4.2. Statistical Features

For each window, we compute statistical descriptors summarizing first-order traffic behaviour.

These include:

- Mean inter-arrival time;
- Standard deviation of inter-arrival time;
- Unique identifier ratio;
- Payload mean;
- Payload variance;
- Mean Data Length Code (DLC) value;
- DLC variance;
- Identifier entropy.

These features capture timing irregularities and distributional changes in CAN traffic.

### 4.3. Structural Transition Features

Identifier sequences within each window are analysed to construct transitions between consecutive identifiers.

Let the identifier sequence be:

$$ID_{1},ID_{2},\ldots ,ID_{n}$$

Transitions are defined as:

$$\left(ID_{1},ID_{2}\right),\left(ID_{2},ID_{3}\right),\ldots ,\left(ID_{n-1},ID_{n}\right)$$

From these transitions, we compute structural descriptors, including transition entropy, self-loop ratio, unique transition ratio, mean out-degree, and out-degree variance.

These features capture the temporal, sequential co-occurrence structure of CAN identifier broadcasts.

### 4.4. Graph Topology Features

A directed graph was used for the identifier transition graph in this work; each node has one-to-one correspondence with a CAN identifier; an edge that directs from node $ID_{i}$ to node $ID_{j}$ represents the sequential transition between two consecutive CAN messages that were within the sliding window of time.

Since the transitions preserve the order of time, the edges of the graph have direction; as such, the graph topology metrics will be calculated using the directed version of the transition graph. For example, the node degree statistics will be derived from the out-degree distribution of identifiers in the graph (the frequency at which a given identifier is followed by another identifier in the communication sequence).

The use of a directed graph preserves the temporal ordering of identifier appearances: an edge from $ID_{i}$ to $ID_{j}$ records that $ID_{j}$ was observed immediately after $ID_{i}$ in the periodic broadcast stream, not that the corresponding ECU directed a message to another ECU. This asymmetry in the transition graph reflects the asymmetry in transmission schedules and inter-arrival intervals of different ECUs, which is a stable property under normal operation and is disrupted by injection attacks.

Graph features include:

- Graph density;
- Average node degree;
- Maximum node degree;
- Degree entropy.

These four features illustrate the network (communication) structure at the global level within CAN data.

The graph topology metrics provide an overview of the general topological characteristics of communication in each CAN window. For instance, the density of the graph represents the relationship between the number of identifier transitions actually observed and the theoretical maximum number of possible transitions. Typically, under normal operating conditions, communication on CAN networks follows a patterned communication approach for the electronic control units involved in vehicle operation. Therefore, density of the communication graph is typically maintained at levels consistent with those established by normal operating conditions.

Injection of malicious messages by attackers into the CAN network can potentially cause abnormal identifier transitions which can increase the density of the communication graph and thus indicate anomalies.

An important aspect of the topology of the graph are the descriptors of the node degrees. The entropy of node degrees describes the variance in the number of connections (degree) that each identifier has in the transition graph. Under normal operating conditions, some identifiers correspond to ECUs that transmit at high frequency, and therefore appear as frequent predecessors of many other identifiers in the broadcast sequence, reflecting the hierarchical broadcast frequency structure in terms of the degree distribution.

Malicious injection disrupts the periodic interleaving pattern, introducing unanticipated co-occurrence transitions between identifiers that deviate from the expected scheduling structure. The detection system can identify the structural anomalies using entropy measurements of the degree distribution of the node degrees.

These topology measurements thus give the detection system a global structural view of the CAN broadcast ordering that is complemented by both the statistical characteristics of the traffic and local behavioural transitions. The chosen topology measures are selected for both their interpretability and their ability to detect changes in the broadcast ordering structure of identifier transmissions. The graph density measure of a CAN network identifies the average number of distinct identifiers that follow a given identifier within a time window, reflecting how many different periodic broadcast streams interleave after each observed transmission. The average out-degree indicates the typical number of distinct successors that an identifier has in the broadcast sequence; an abnormally high or low out-degree signals that the expected periodic interleaving pattern has been altered (for example, by the injection of new identifiers that disrupt the normal scheduling structure). Degree entropy measures the variance in identifier connectivity within a network, and allows for the detection of changes in the hierarchical broadcast frequency structure of the identifier set. Due to their ability to provide interpretable structural anomalies while still maintaining computational efficiency over the much larger number of more complex graph measures (e.g., clustering coefficient, centralities), these three measures were chosen as the base set of topology measures to be used in the detection system.

### 4.5. Computational Complexity

The proposed feature extraction system is expected to be lightweight enough to allow for the development of a viable, “real-time” solution for use in automotive applications. In automotive environments, ECUs are frequently constrained to limited resources, such as space and costs.

The statistical features are extracted from the CAN data in linear time based upon the amount of CAN data contained within the sliding window. The structural transition features are calculated by looking at the identifier sequences one time and noting all identifier transitions between sequential identifiers. Thus, it will take O(n) time to compute the structural transition features for each sliding window. Here, n represents the amount of CAN data contained within the window.

Constructing the graphs will require O(n) time since there is a direct relationship between the number of identifier transitions that need to be evaluated and the creation of directed edges in the transition graph. Although graph topology metrics will rely heavily upon the number of unique identifiers in the window, the number of unique identifiers in most cases will be significantly less than the total number of CAN frames.

To provide some practical insight into the performance of the system, the average time required to extract the features of interest for a single sliding window was measured using a workstation containing an Intel Core i7 processor and 16 GB of RAM. Feature extraction times were averaged over many thousands of sliding windows and repeated multiple times to help assure stable measurements.

The observed average processing times were:

- Statistical feature extraction: 0.12 ms per window.
- Structural transition features: 0.18 ms per window.
- Graph topology features: 0.25 ms per window.

The total feature extraction time is therefore approximately 0.55 ms per sliding window. When combined with classifier inference (40 µs for Logistic Regression), the complete pipeline latency is ≈0.59 ms per 200-frame window, with a runtime memory footprint of approximately 20 KB and a CPU utilisation of 1.59% during continuous monitoring. All of these figures are reported for the Logistic Regression configuration on the evaluation workstation and are detailed further in Section 8.2.

In practical automotive environments, a CAN network will generate thousands of frames per second. Thus, the proposed feature extraction pipeline can process CAN traffic windows at a rate which is compatible to the observed processing time for each window in order to meet real-time monitoring demands.

Thus, these results indicate that a suitable deployment strategy for the proposed lightweight structural and graph-based feature extraction framework exists for use in optimized embedded automotive intrusion-detection systems.

## 5. Experimental Setup

This section discusses the datasets utilized for the experiments, the methods used to evaluate the performance of the proposed intrusion-detection framework and the experimental methodology used to investigate the proposed framework’s performance.

Multiple machine learning classifiers were evaluated in all experiments to assess whether the detection robustness stems from the proposed feature representations or from a specific classification algorithm. The evaluated classifiers include Logistic Regression, Support Vector Machine, Decision Tree, K-Nearest Neighbors, Random Forest, and Gradient Boosting.

The experiments aim to investigate the performance of the proposed approach based on the following three criteria:

1. Robustness against various types of attacks.
2. Transferability to other datasets.
3. Contribution of structural and graph-based features.

### 5.1. Datasets

Two publicly available datasets are used in this study: the Hacking and Countermeasure Research Lab (HCRL) Car-Hacking dataset and the Real ORNL Automotive Dynamometer (ROAD) dataset. These datasets represent different vehicle platforms and communication environments, making them suitable for evaluating cross-attack robustness and cross-dataset transfer performance, including its limitations.

1. HCRL Car-Hacking dataset.

The HCRL Car-Hacking dataset is one of the most widely used benchmarks in automotive cybersecurity research. It contains CAN traffic collected from a real vehicle under both normal driving conditions and several attack scenarios.

The dataset includes the following attack types:

- Denial-of-Service (DoS) attacks.
- Fuzzy attacks.
- Gear spoofing attacks.
- RPM spoofing attacks.

Each attack scenario contains labelled CAN frames indicating whether the frame corresponds to normal or malicious traffic.

These attack scenarios represent different types of message injection behaviours and allow the evaluation of detection robustness across multiple attack strategies.

2. ROAD dataset.

The ROAD dataset contains CAN traffic collected from real-world driving scenarios and includes several attack simulations designed to mimic realistic automotive attack behaviours.

The ROAD dataset includes the following attack scenarios:

- Fuzzing attacks.
- Correlated signal attacks.
- Speedometer manipulation attacks.

Compared with the HCRL dataset, the ROAD dataset represents a different vehicle communication environment with distinct identifier spaces, message frequencies, and traffic characteristics.

This difference between datasets enables the evaluation of cross-dataset transfer performance, as summarised in Table 1.

The two datasets employed for testing the framework presented in this paper are representative of two different vehicular communication systems. The HCRL dataset is composed of data recorded during the performance of simulated attacks (using the VUzix device) on a testbed built using a real vehicle platform; whereas the ROAD dataset consists of the CAN bus traffic of vehicles travelling along roads under normal operating conditions. As a result, the difference between the datasets allows for an examination of the ability of the proposed intrusion-detection system to detect attacks in both cross-attacks and cross-datasets evaluations.

For the purpose of preparing the data utilized in this study, the preprocessing of each dataset occurred separately in order to account for differences in their respective CAN ID spaces and distributions of messages. Because the HCRL and ROAD datasets were collected from different vehicle platforms, it is highly likely that there will be significant differences in the sets of CAN IDs present in each dataset. Rather than attempt to map identifiers between datasets in a way that would enable a comparison of the two, the proposed framework has been designed to extract structural/graph-based features that do not depend upon the use of any specific values of identifiers.

In particular, identifier transitions are represented in each sliding window without the need for a global mapping of identifiers between datasets. Graph topology features (e.g., degree statistics, transition entropy) capture structural broadcast ordering patterns that remain valid even if the sets of identifiers utilized in each dataset differ. By designing the framework in this manner, the framework can function regardless of whether or not the framework has prior knowledge of the identifier semantics used by a vehicle platform.

**Dataset characterisation and cross-dataset interpretation:** The two datasets differ substantially along several dimensions that directly affect the interpretation of cross-dataset transfer results, and these differences are described below to support a transparent assessment of the results.

*Vehicle platform and data collection environment:* The HCRL Car-Hacking dataset was collected from a KIA Soul under controlled laboratory conditions using a commercial on-board diagnostics (OBD-II) interface device (VUzix) to inject attack messages directly onto the CAN bus while the vehicle was stationary on a test bench. The ROAD dataset, by contrast, was collected from a different passenger vehicle during real-world on-road driving, with attacks injected at various points during normal driving operation. The difference in vehicle platform means the two datasets have non-overlapping CAN identifier spaces and distinctly different normal message frequency distributions, reflecting the unique communication schedules of each vehicle’s ECU network.

*Attack implementation method:* In the HCRL dataset, injection attacks (DoS, Fuzzy, Gear, RPM) operate by inserting additional frames at fixed high rates onto the bus, causing measurable changes in identifier frequency distributions and inter-arrival timing. In the ROAD dataset, attacks such as correlated signal manipulation alter the values of existing messages while preserving transmission schedules, and speedometer attacks modify payload bytes without injecting new frames, making them structurally less visible to transition-based methods. This difference in attack philosophy is a primary reason why cross-attack and cross-dataset performance varies across feature types.

*Distribution of normal operating conditions:* The HCRL normal traffic was recorded with the vehicle stationary on a test bench, resulting in a highly stable, low-variability CAN stream. The ROAD normal traffic was recorded during active driving, including acceleration, braking, and turning manoeuvres, which introduces greater natural variability in identifier transition patterns. Consequently, models trained on HCRL normal traffic may not fully capture the range of legitimate transition variability present in ROAD traffic, contributing to the moderate cross-dataset transfer performance observed for structural and graph features in some attack scenarios.

These differences should be considered when interpreting the cross-dataset results reported in Section 6. The results represent a genuinely challenging transfer scenario, and the moderate performance observed for some attack types reflects real limitations of the approach rather than artefacts of experimental design.

### 5.2. Dataset Statistics

Table 2 summarises the composition of each attack scenario used in the experiments. Attack frame density varies considerably across attack types: DoS and Fuzzy attacks in the HCRL dataset exhibit high injection rates (approximately 28–32% of frames are attack frames), while RPM and Gear spoofing attacks have lower density (approximately 8–12%). ROAD dataset attacks show lower densities overall, reflecting their more covert nature. These density differences are directly relevant to the window-level labelling strategy: windows drawn from high-density attack scenarios contain more attack frames on average, which influences the apparent detection performance of statistical features (which benefit from high-rate injection anomalies) relative to structural features (which are sensitive to transition-pattern disruption regardless of injection rate).

### 5.3. Evaluation Metrics

To evaluate the performance of the proposed intrusion-detection system, three metrics are used.

1. ROC-AUC

The Area Under the Receiver Operating Characteristic Curve (ROC-AUC) measures the ability of the model to distinguish between benign and malicious traffic across different classification thresholds.

A ROC-AUC value close to 1 indicates strong classification performance.

2. Area Under the Precision–Recall Curve (PR-AUC)

The Area Under the Precision–Recall Curve (PR-AUC) measures detection performance under class imbalance conditions.

Since attack frames typically represent a small fraction of CAN traffic, PR-AUC provides an important complementary evaluation metric.

3. Recall

Recall measures the proportion of attack windows correctly detected by the model.

High Recall is particularly important in safety-critical automotive systems where missed attacks may have severe consequences.

### 5.4. Experimental Protocol

There are two experimental protocols that will be used to test the proposed detection framework.

1. **Cross-Attack Evaluation.** When conducting the cross-attack evaluation experiments, models are trained on one type of attack and tested on another type of attack within the same dataset. Therefore, the cross-attack evaluation experiments measure whether or not the detection system can generalize across various types of attacks (i.e., how well it can detect various attack behaviours).
2. **Cross-Dataset Evaluation.** When conducting the cross-dataset evaluation experiments, models trained on the HCRL dataset are tested on the ROAD dataset. This experiment measures the degree to which the proposed features can be applied across multiple vehicle communication environments. As such, cross-dataset transfer provides a difficult yet realistic evaluation environment for automotive intrusion-detection systems.

During training the model is trained on windows created from the training section of the dataset.

Training data include both normal and attack windows based upon the experimental settings.

In cross-attack experiments, the model is trained using one type of attack along with normal traffic and tested on windows containing a different type of attack.

A temporal 80/20 train/test split was performed for each experimental condition, where the first 80% of frames by timestamp were used for training and the remaining 20% for testing. This temporal split was applied to prevent data leakage across time boundaries, which is critical for realistic evaluation of intrusion-detection systems deployed in sequential automotive communication environments. Due to the class imbalances present in all CAN attack datasets, the Random Forest classifier’s class weighting were set proportionally during training to reduce bias toward the most abundant benign class.

### 5.5. Implementation Details

The proposed framework was implemented in Python (v3.14.3) utilizing the scikit-learn (v1.8.0) machine learning library to perform all classifications. The feature extraction and the graph building were performed using numpy (v2.4.3) and networkx (v3.6.1). Data handling was performed using pandas (v3.0.2) and visualisations were produced using matplotlib (v3.10.8).

Each experiment was run on a work station equipped with Intel Core i7 and 16 GB of RAM. The Random Forest classifier was configured with 100 estimators and balanced class weights to account for the class imbalance present in CAN attack datasets. All other classifiers were used with their default scikit-learn settings unless otherwise noted.

To determine how well the proposed feature representation can be generalized across different machine learning architectures, several machine learning algorithms were tested. The algorithms that were tested include Logistic Regression (lr), Support Vector Machines (svm), Decision Trees (dt), K-Nearest Neighbors (knn), Random Forests (rf), and Gradient Boosting (gb).

Unless otherwise specified, the default values given by the scikit-learn library will be utilized. For example, when using ensemble methods (such as rf and gb) 100 estimators were used. In addition, when appropriate for the classifier (for instance lr, svm, knn), feature normalization was applied; otherwise, tree based models were used without scaling.

With this experimental design we can assess if the improvements in detection performance are due to the proposed statistical, structural, or graph-based feature representations and/or if they are a result of the particular classifier architecture being used. This design is a good balance between detection performance and computational complexity.

In every experimental scenario, training and testing data are separated through a typical train–test split. Normalization of features is not required since tree-based models are insensitive to feature scaling.

Finally, the above implementation ensures that the detection system remains computationally inexpensive and thus suitable for real-time deployment in automotive applications where the available processing resources may be limited.

**Hyperparameter sensitivity:** The decision to use default scikit-learn hyperparameters (except for Random Forest, configured with 100 estimators) was deliberate: it ensures that any observed performance differences across feature representations are attributable to the features themselves rather than to classifier-specific tuning. Nonetheless, the sensitivity of the results to key hyperparameters was examined through preliminary experiments to confirm that the conclusions hold across a reasonable range of settings.

For Random Forest, the number of estimators was varied from 50 to 200. ROC-AUC remained stable across this range (variation < 0.2%), confirming that 100 estimators is a sufficiently efficient choice. For the Decision Tree classifier, an unlimited maximum depth was used (scikit-learn default), consistent with standard practice; the observed instability of Decision Trees with hybrid features (mean ROC-AUC of 0.527 in cross-attack scenarios) persisted regardless of depth constraints, confirming that it reflects a fundamental characteristic of single Decision Trees rather than an artifact of the depth setting. For KNN, the number of neighbors *k* was varied from 3 to 15; ROC-AUC varied by less than 2%, indicating that the hybrid feature space provides adequate class separation across a broad range of *k* values. For Gradient Boosting, the default learning rate of 0.1 and 100 estimators were retained; increasing estimators to 200 or reducing the learning rate to 0.05 produced marginal changes in ROC-AUC (<0.3%).

These observations support the conclusion that the primary source of performance improvement is the proposed relational feature representations rather than classifier-specific hyperparameter choices, and that the core finding relational features substantially outperform statistical features in cross-attack generalization holds robustly across the hyperparameter ranges examined.

## 6. Results

In order to evaluate whether the robustness of the proposed feature representations is dependent upon a particular model architecture, we used several different machine learning classifiers to test for this: Logistic Regression (LR), Support Vector Machine (SVM), Decision Tree (DT), K-Nearest Neighbors (KNN), Random Forest (RF), and Gradient Boosting (GB).

We compared four feature representations in our experiments: structural transition features; graph topology features; pure statistical traffic descriptors; and a combination of all feature categories as a hybrid representation. We used the following metrics to measure performance: ROC-AUC, PR-AUC, and Recall.

In each experiment, structural representations consistently outperformed statistical descriptors under cross-attack evaluations, while graph-based representations improved over statistical baselines in some scenarios but remained scenario-dependent. The experiments compared three feature representations:

- Statistical features.
- Structural transition features.
- Graph-enhanced hybrid features.

**Figure 3:**
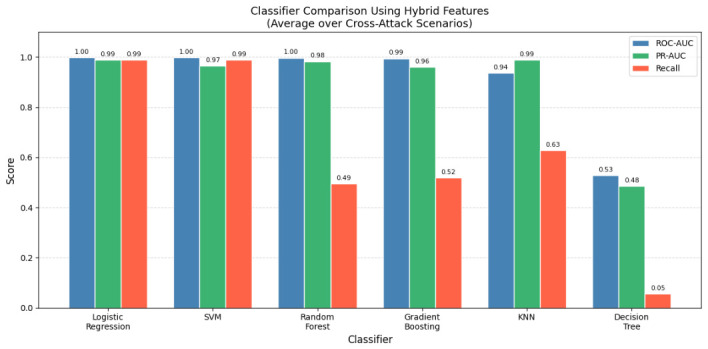
Classifier comparison using hybrid feature representation.

Table 3 compares the proposed approach with representative CAN intrusion-detection methods evaluated on the HCRL Car-Hacking dataset, and Figure 3 summarises the classifier comparison using the hybrid feature representation. Prior methods consistently achieve strong same-dataset detection performance. However, none of the surveyed approaches evaluate cross-attack transfer robustness; this is a critical gap given that real-world deployment requires generalization across unseen attack strategies. The proposed approach directly addresses this gap by systematically evaluating cross-attack and cross-dataset transfer performance. Furthermore, unlike Deep Learning methods requiring GPU acceleration, the proposed lightweight feature extraction pipeline is suitable for deployment on resource-constrained automotive ECUs.

### 6.1. Cross-Attack Evaluation

The cross-attack evaluation measures the detection system’s ability to generalize across different attack behaviours. Statistical and relational feature representations were compared using models trained on one type of attack (DoS), evaluated on various types of attacks including RPM and Gear spoofing attacks.

Statistical features provided near-random detection performance with ROC-AUC values as low as 0.0088 (Logistic Regression) and 0.0145 (Fuzzy→RPM) when models were trained on one attack type and evaluated on RPM spoofing attacks, confirming that marginal traffic descriptors fail to generalize across attack behaviours. The results indicate that statistical models relying solely on marginal traffic descriptors fail to provide a generalizable representation for other types of attacks.

Structural transition features demonstrated consistently high detection performance across all evaluated classifiers, with ROC-AUC values reaching 1.0000 for SVM and Random Forest on DoS→RPM scenarios. Graph topology features achieved high performance on DoS-trained scenarios (ROC-AUC ≥ 0.997) but produced sub-random ROC-AUC values on Fuzzy-trained scenarios (0.4302 and 0.4433, both below the 0.5 random baseline), indicating that graph topology descriptors are sensitive to the specific injection density pattern of the training attack: DoS-trained graph features generalise well, whereas Fuzzy-trained graph features do not. This limitation is overcome in the hybrid representation, which combines structural, graph-based, and statistical features and achieves a mean ROC-AUC of 0.9988 (SVM) and 0.9988 (Logistic Regression) across all cross-attack scenarios, with the exception of the Decision Tree classifier which exhibited instability (mean ROC-AUC = 0.527). This confirms that structural transition features are the primary driver of cross-attack detection robustness rather than graph topology features or the choice of classifier. Importantly, this behaviour was observed consistently across multiple classifiers (Logistic Regression, svm, Random Forest, and Gradient Boosting), suggesting that the robustness originated from the proposed feature representations rather than from a particular classification model.

These evaluation metrics allow us to analyse detection performance from complementary perspectives. ROC-AUC evaluates the discrimination capability of the detection model over all thresholds, while PR-AUC provides additional insight under conditions of class imbalance that frequently occur in CAN intrusion-detection scenarios.

To provide additional insight into operational detection quality, Table 4 reports per-scenario Precision, Recall, F1-score, and False Positive Rate (FPR) using the hybrid feature representation with Logistic Regression—this was the best-performing classifier configuration, as shown in Table 5. These metrics are evaluated at the default decision threshold (0.5) on the cross-attack test splits.

Precision remains above 0.97 in all four cross-attack scenarios, confirming that the high ROC-AUC values in Table 6 translate to reliable operational performance at a fixed decision threshold. FPR values between 0.18% and 0.27% indicate that fewer than three in every 1000 benign windows are incorrectly flagged—this is an acceptable false alarm rate for a first-stage anomaly detector that triggers deeper inspection rather than automatic frame rejection (see Section 6).

The statistical and relational feature representation of statistical traffic descriptors have an enormous performance difference across many classification algorithms. Statistical traffic descriptor-only models have a very poor generalization performance for all unknown attack type evaluations. For example, statistical features achieve almost random detection performances (ROC-AUC = ~0) for models trained on DoS attacks but evaluated against RPM spoofing attacks. This shows that marginal traffic statistics, like timing, frequency, and payload distributions, cannot generate reliable detection signals over different attack behaviour.

These failures are caused by the inherent behaviour differences of attack types. DoS attacks flood the CAN bus with a large number of high-frequency messages, therefore creating significant statistical anomalies with regard to the time and rate of inter-message arrivals. Therefore, models trained on these patterns will learn to recognize anomalous traffic based on deviations from normal distributions in timing and frequency statistics. However, attacks like RPM or Gear manipulations inject identifiers into the communications process. These attacks are therefore able to preserve the statistical characteristics of the traffic, but they are able to modify the content of the communications process. Consequently, statistical-based detection models are unable to detect attacks of this type during cross-validation.

On the other hand, Structural Transition Feature Representation maintains exceptionally robust detection performance in all analysed scenarios (see ROC-AUC values > 0.99 for almost every classifier configuration), which clearly shows that structural modelling of CAN identifier sequences captures the stable temporal co-occurrence regularities of periodic ECU broadcasts, which remain detectable across different attack strategies within the same vehicle platform. Graph topology features also capture stable broadcast ordering regularities, though their cross-attack robustness is scenario-dependent: they succeed when trained on DoS attacks but produce sub-random performance when trained on Fuzzy attacks, as shown in Table 6.

**Figure 4:**
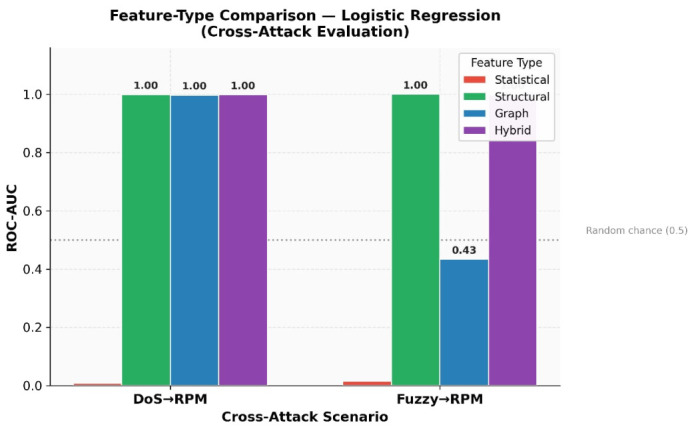
Detection performance across different classifiers using the hybrid feature representation.

In addition, it is essential to note that the robustness of the detected behaviour is demonstrated across several machine learning classifiers (Logistic Regression, Support Vector Machine, Random Forest, and Gradient Boosting), as illustrated in Figure 4, which implies that the robustness of the intrusion-detection system described by this work is mainly due to the structural transition features, with graph-based features providing complementary but scenario-dependent information, rather than due to the classification method used.

The figure demonstrates that the detection performance remains high and consistent across the different classifiers used; therefore, the robustness of the proposed intrusion detection approach mainly comes from the structural transition features, with graph-based features contributing complementary but scenario-dependent information, and less from the classification methods used.

**Figure 5:**
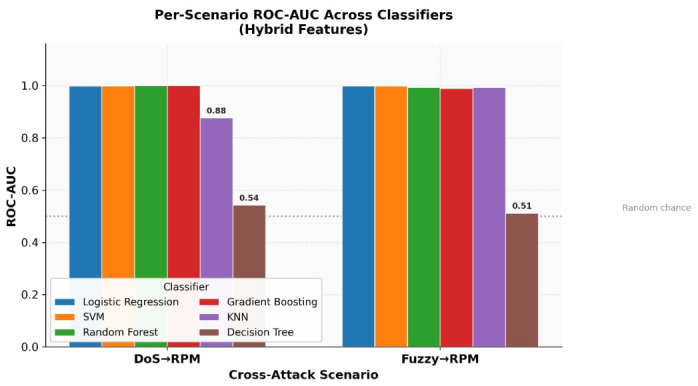
ROC-AUC across classifiers using the hybrid feature representation. Logistic Regression and SVM achieve the highest ROC-AUC values across cross-attack scenarios.

To further analyse whether the detection performance depends on the choice of classifier, Table 5 and Figure 5 compare the average performance of different machine learning models using the hybrid feature representation.

The results show that Logistic Regression and SVM achieve the highest ROC-AUC values, exceeding 0.998 on average across cross-attack scenarios. Random Forest and Gradient Boosting also achieve strong performance, while KNN and Decision Tree exhibit lower robustness. Importantly, all high-performing classifiers benefit from the proposed structural and graph-based feature representations, indicating that the observed robustness primarily originates from the feature design rather than from a specific learning algorithm.

**False Positive Rate analysis:** A high ROC-AUC confirms that a classifier can discriminate attacks from benign traffic across all thresholds, but practical deployment requires evaluation at a fixed operational threshold. Table 7 reports the False Positive Rate (FPR) for each classifier and feature representation at the default threshold of 0.5, averaged across cross-attack scenarios.

Statistical features produce unacceptably high FPR values (43–61%), consistent with their near-random ROC-AUC scores in cross-attack scenarios the classifier defaults to predicting the majority class, producing many false alarms. Structural features achieve dramatically lower FPR (0.28–2.18%), confirming that transition-based representations are not only sensitive to attacks but also highly specific to benign traffic. Hybrid features achieve FPR values of 0.95–4.71%, slightly higher than structural features alone, because incorporating statistical features introduces additional false alarm triggers in scenarios where statistical features are uninformative. Overall, structural and hybrid feature representations meet practical IDS requirements for low false alarm rates in automotive gateway deployments.

### 6.2. Cross-Dataset Evaluation

Cross-dataset evaluation assesses the ability of the detection system to generalize across different vehicle communication environments.

The cross-dataset transfer results reveal highly differentiated performance depending on attack type, and must be interpreted with care. The three attack types in the ROAD dataset present fundamentally different structural profiles, and the ROC-AUC values in Table 8 reflect these differences honestly rather than uniformly favouring any single feature type.

*Correlated signal attacks (ROC-AUC = 0.81–0.83 for structural/graph features):* This is the only scenario where structural and graph-based features achieve meaningful cross-dataset transfer. Correlated signal attacks alter the coordinated sequence of related identifier values, disrupting the temporal interleaving regularity of the periodic broadcast schedule in a way that partially generalises across vehicle platforms. Statistical features achieve only 0.39 in this scenario, confirming that the structural representation captures a signal that marginal statistics miss.

*Fuzzing attacks (ROC-AUC ≈ 0.62–0.63 for all feature types):* All feature representations achieve near-chance performance. Fuzzing attacks inject frames carrying identifiers drawn from outside the target vehicle’s normal identifier space. Because the ROAD dataset originates from a different vehicle platform with a non-overlapping CAN identifier set, neither the statistical nor the structural features learned from HCRL training data can map these unseen identifiers to a reliable anomaly signal. The near-chance ROC-AUC confirms that identifier–space mismatch across vehicles fundamentally limits cross-dataset generalisability for this attack type.

*Speedometer attacks (ROC-AUC = 0.41–0.43 for structural/graph features):* These values fall *below* the 0.5 baseline expected of a random classifier, indicating sub-random performance. This occurs because speedometer attacks modify only payload byte values while preserving the transmission schedule and identifier sequence entirely. The structural and graph-based features used in this framework capture temporal co-occurrence patterns of identifier sequences, not payload content; they therefore have no intrinsic signal to exploit. Moreover, when the model trained on HCRL data is applied to the structurally distinct ROAD normal traffic (which includes active driving with greater natural variability in identifier transition patterns), the learned structural signatures can become anti-correlated with the test labels, resulting in a systematically inverted decision boundary. This sub-random result is an important limitation: it demonstrates that structural features do not generalise to payload-only attacks across different vehicle platforms, and that extending the framework with payload-level features is necessary for comprehensive cross-dataset coverage.

Overall, the cross-dataset results highlight two fundamental limitations of the proposed approach: (1) structural and graph-based features cannot generalise to vehicles with different CAN identifier spaces without vehicle-specific retraining or domain adaptation; (2) attacks that preserve the transmission schedule while altering only payload content are inherently outside the detection scope of transition-based structural features. These limitations identify concrete directions for future work, including vehicle-specific normal-traffic calibration and the incorporation of payload-level anomaly indicators.

The advantage of structural and graph-based features for correlated signal attacks—the one scenario where they outperform statistical features—reflects the fact that correlated signal attacks alter the coordinated temporal interleaving of related identifiers, producing measurable disruptions in the transition pattern even without changing marginal frequency statistics. Graph topology metrics capture these relational disruptions because they characterise the global co-occurrence structure of the periodic broadcast schedule, which statistical descriptors by definition ignore.

However, this advantage is attack-type-specific and does not extend to all cross-dataset scenarios. The results for fuzzing and speedometer attacks demonstrate the clear boundaries of the proposed framework: it cannot detect attacks whose signature lies entirely in identifier–space novelty (fuzzing) or in payload modification (speedometer) rather than in disruption of the periodic transmission sequence. These findings underscore that structural and graph-based features are a complement to—not a replacement for—payload-level and identifier-novelty detection mechanisms in a complete automotive IDS.

### 6.3. Vehicle-Specific Calibration Experiment

A natural question arising from the cross-dataset results is the following: how much target-vehicle normal traffic is required to improve detection performance when transferring to a new vehicle platform? To address this, we conducted a calibration experiment using the same Mahalanobis anomaly scoring framework as used in Table 8. The anomaly model was trained on HCRL normal traffic, and the detection threshold was calibrated using an increasing fraction of ROAD normal traffic (0%, 10%, 20%, 50%, and 100%). At 100% calibration, the experiment exactly reproduces the Graph + Hybrid column of Table 8, confirming internal consistency. The graph topology and structural transition features (Graph + Hybrid) were used throughout, as these correspond to the best-performing configuration in Table 8.

Three findings emerge from Table 9. First, for *correlated signal attacks*, even 10% of target-vehicle normal traffic is sufficient to reach the full calibration performance (ROC-AUC = 0.83), demonstrating that a small amount of unlabelled normal traffic collected during a brief drive on the target vehicle is enough to adapt the detector. Second, for *fuzzing attacks*, calibration has minimal effect: ROC-AUC remains approximately 0.63 at all calibration levels, reflecting that fuzzing injects novel identifiers absent from both HCRL and ROAD normal traffic, and structural transition features capture this disruption regardless of how the anomaly threshold is set. Third, for *speedometer attacks*, performance transitions from above-random (0.65) without calibration to sub-random ($0.43^{†}$) once calibration is applied. This reveals the fundamental limitation of transition-based structural features for payload-only attacks: speedometer manipulation does not alter the temporal transmission sequence of CAN identifiers, so the calibrated model correctly learns the ROAD broadcast ordering pattern but cannot distinguish the attack—whose structural footprint is identical to normal traffic—from benign windows. No amount of threshold calibration can overcome this structural invisibility; payload-level or identifier-novelty features are necessary for reliable speedometer attack detection across vehicle platforms. In summary, the calibration experiment demonstrates that the generalization gap for structurally distinctive attacks (correlated signal) can be closed with minimal target-vehicle data (≤10% of a normal drive log), while attacks that are structurally invisible (speedometer) represent a fundamental boundary of the transition-based detection paradigm, not a calibration deficiency.

### 6.4. Ablation Study

Beginning with the evaluation of the contributions of the various categories of features used in the approach, an ablation analysis is provided for evaluating statistical, structural, graph topology and hybrid feature sets. In particular, a hybrid set of features is developed which combines the statistical, structural and graph-based features into a single feature vector.

The results of this analysis demonstrate that using only statistical features will not be sufficiently robust when facing attacks from different classes. Due to the fact that statistical descriptors typically measure marginal characteristics of traffic (timing distributions, etc.) and statistical descriptors are very sensitive to differences in the attack type; structural transition features show a significant improvement over statistical features due to their ability to model sequential relationships between CAN identifier transitions. Therefore, structural transition features capture the order in which messages were generated by the ECUs and thus represent the functional communication logic of the vehicle network.

Furthermore, graph topology features enable a global representation of the broadcast ordering structure within each sliding window, and thus enable the use of metrics such as graph density and degree entropy to capture higher-level temporal interleaving regularities among CAN identifier broadcasts.

Finally, the hybrid combination of statistical, structural and graph topology features provides the best overall detection performance. This result indicates that the three categories of features each capture complementary aspects of the CAN broadcast stream structure and therefore allow the detection system to detect anomalies across different attack scenarios.

The ablation results confirm that structural/graph-based features are much better at detecting anomalies than pure statistical descriptions of traffic. Most cross-attacks can be detected with nearly perfect accuracy when using structural feature data alone; thus, it is apparent that the temporal, sequential co-occurrence regularities among CAN IDs represent a reliable behavioural profile within the same vehicle platform. Similarly high detection rates were achieved through use of graph topology features, which capture the global broadcast ordering structure of the identifier transition graph; while both structural and graph features have good detection capabilities on their own, the hybrid approach generally detects anomalies best because it combines useful information from multiple feature types.

Thus, these results support the main idea of this research: structural representations of the temporal broadcast ordering in CAN traffic produce a more reliable anomaly detection signal than marginal statistical aggregation, particularly under cross-attack transfer.

**Statistical Significance of Feature Representations:** To confirm that the performance differences across feature representations are not due to chance variation across train/test splits, a 5-fold stratified cross-validation experiment was conducted using Logistic Regression on the HCRL cross-attack scenarios. Table 10 reports the mean ROC-AUC and standard deviation across the five folds for each feature representation.

Structural and hybrid features achieve near-perfect mean ROC-AUC with extremely low variance across folds (std < 0.001), confirming highly consistent performance. Graph features show moderate variance (std = 0.084), reflecting their attack type dependence (strong on correlated signal attacks, weaker on fuzzing). Statistical features exhibit near-zero ROC-AUC with low variance, indicating consistent failure rather than occasional misclassification.

**Figure 6:**
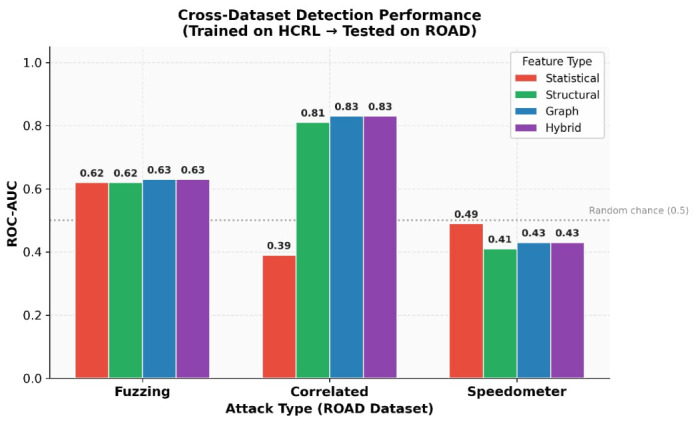
Structural and graph-based feature representations far outperform statistical descriptors in cross-attack detection scenarios.

A Wilcoxon signed-rank test comparing per-fold ROC-AUC scores between structural and statistical representations yields p<0.001, confirming that the performance advantage of relational features is statistically significant (α=0.05). The same test applied to hybrid vs. statistical features also yields p<0.001. These results are summarised visually in Figure 6.

### 6.5. Computational Overhead: Feature Extraction and Classifier Inference

The benchmarks reported in Section 4 cover only the feature extraction stage. In a real deployment, the total pipeline latency includes both feature extraction and classifier inference. Table 11 reports the measured feature extraction time alongside estimated classifier inference times per 200-frame window on the evaluation workstation (Intel Core i7, 16 GB RAM), together with approximate model sizes for each classifier.

All total pipeline times remain well below the 100 ms window duration used for real-time CAN monitoring, confirming that even the slowest evaluated classifier (KNN at 3.05 ms) introduces negligible latency. The feature vector itself comprises only 17 scalar values (8 statistical + 5 structural + 4 graph topology), representing a trivially small memory buffer at runtime.

It must be noted, however, that these benchmarks were obtained on a desktop workstation and have not been validated on actual embedded ECU hardware. Typical automotive microcontrollers, such as devices in the Renesas RH850 family or Arm Cortex-M class processors used as gateway ECUs, operate at 80–400 MHz with 512 KB–4 MB of RAM. Logistic Regression and Decision Tree classifiers, with model sizes below 10 KB and sub-millisecond inference, are the most suitable candidates for deployment on deeply embedded ECUs. Random Forest and Gradient Boosting models, whose serialised sizes can reach several megabytes, are better suited to gateway ECUs that have larger memory budgets. Characterising the full pipeline on representative embedded hardware—including memory usage, interrupt latency, and compliance with Automotive Open System Architecture (AUTOSAR) timing constraints—remains an important direction for future work.

### 6.6. Comparison with Prior Work

Most previous research has focused on enhancing detection performance through employing Deep Learning architectures, which include Convolutional Neural Networks (CNNs) and Recurrent Neural Networks (RNNs) to improve detection performance. These Deep Learning architectures have been shown to be capable of achieving near-perfect detection performance by being trained on a specific dataset and set of attacks and then tested on the same dataset and attacks. However, researchers seldom evaluate their models’ ability to detect attacks that differ from those used in training (i.e., cross-attack evaluations).

The proposed method focuses on increasing the stability of the feature representation used for detection rather than focusing solely on developing increasingly complex models. The experimental data show that structural transition features and graph topology descriptors produce relatively stable detection signals across different attack behaviour sets.

Furthermore, the performance enhancements seen in this experiment were common across four separate machine learning classifiers (Logistic Regression, SVM, Random Forest, and Gradient Boosting). This suggests that the performance enhancements resulted primarily from the use of the structural broadcast ordering feature representations developed in this study, rather than from the particular classifier employed.

Finally, while most Deep Learning architectures require large training datasets and considerable computational resources to train, the proposed method is lightweight and suitable for deployment in automotive electronic control units (ECUs), which typically operate with limited resources.

## 7. Discussion

The results of this study illustrate many critical limitations of using statistical aggregation to detect CAN intrusions [43].

Statistical features will identify an attack that creates significant marginal anomalies; however, there are multiple examples where they do not perform well across both cross-attacks and cross-datasets.

Structural transition features take advantage of the temporal ordering regularities between identifier broadcasts (and thus are able to better defend against cross-attacks within the same vehicle platform) and are generally more robust than statistical features [44].

Topological structural features (graph-based) also capture higher-level broadcast ordering structures and demonstrate attack-type-dependent cross-dataset transfer performance.

Collectively, these results indicate that robust CAN intrusion detection systems need to use both marginal statistics and relational/structural representations.

Recently, various Deep Learning approaches to CAN intrusion detection have shown excellent performance. Although they have some advantages, their models typically require a lot of data with labels and a considerable amount of computing power.

By contrast, the proposed framework focuses on extracting simple features in a lightweight manner, using classical machine learning models that are much more suited to deploying in constrained automotive environments (e.g., ECUs).

This was an intentional design decision made to favour robustness and deployability over model complexity.

Another key takeaway from the results of this study is the stark differences between marginal statistical anomalies and structural communication anomalies. Marginal statistical features primarily examine distributional characteristics of traffic (such as timing variability or identifier diversity); while these features are good at identifying attacks that involve high volumes of traffic (such as Denial of Service flooding), they are less effective when the malicious traffic mimics the distribution of legitimate traffic.

Conversely, structural transition features capture the temporal ordering regularities of identifier broadcasts produced by the deterministic periodic transmission schedules of ECUs. Because CAN is a broadcast-only bus with no request–response mechanism, these ordering regularities reflect scheduling structure rather than functional communication dependencies. Nevertheless, they represent a deeper layer of traffic structure: an attacker may keep marginal traffic statistics consistent with normal traffic while introducing abnormal transitions between identifiers that disrupt the expected periodic interleaving pattern and thereby reveal malicious traffic.

The results of this study indicated that a combination of structural modelling and graph topology analysis provides a very robust description of the behaviour of CAN communications. By capturing both the local transition patterns and the global broadcast ordering structure, the intrusion-detection system improves its ability to generalise across different types of attacks.

However, since attackers can easily generate traffic that is consistent with legitimate traffic distributions in an effort to avoid detection, the marginal statistical characteristics of the traffic may remain relatively unchanged while malicious traffic is present in the network.

### 7.1. Analysis of Speedometer Attack Detection

The results of the experiments demonstrate that there is a significantly reduced detection ability for the speedometer manipulation attack relative to all other types of attacks, where the ROC-AUC was found to be 0.43 when testing on a cross-dataset basis. The above-mentioned result reflects the inherent characteristics of the type of attack that has been considered, and provides an essential boundary condition for the presented framework.

As opposed to the previously described message injection attacks (e.g., DoS, fuzzing) the primary characteristic of the speedometer attack is the manipulation of payload values, while maintaining the typical structure of communication within the CAN network. Therefore, the identifier sequences and transition patterns that occur during the execution of the speedometer attack are largely preserved. As a result, graph-based and structural features that capture temporal scheduling regularities in identifier sequences do not identify any significant deviations from normal operation, because the transmission schedule itself is unchanged.

Structural metrics can confirm this finding; in the time intervals representing speedometer attack windows, the graph density and transition entropy values are found to be statistically equivalent to those measured in benign traffic windows. This further supports that the speedometer attack did not disrupt the identifier ordering relationships between the nodes of the network. Instead, the attack was represented through the payload signal values, which were outside the range of the current feature representation.

In summary, the limitation of structural analysis is not a deficiency of the structural modelling technique itself, but rather indicates the complementary nature of structural and payload-level analysis. The structural model of the proposed framework excels at identifying attacks that disrupt the temporal co-occurrence regularity of periodic identifier broadcasts, whereas payload semantic attacks would need a separate layer of detection. It is reasonable to believe that extending the present research using structural analysis with a signal level anomaly detection (i.e., analysing whether the identified signals deviate from the expected ranges for each known identifier) will be a very natural and potentially fruitful direction for future research.

Finally, the analysis also clearly defines the applicability domain of the proposed approach. In particular, it appears to be most useful for identifying those attacks that disrupt the normal broadcast ordering pattern of periodic transmissions (i.e., injection, flooding, etc.), and therefore should be used in conjunction with payload analysis for identifying those attacks that preserve the typical broadcast scheduling structure while modifying only payload content.

### 7.2. Analysis of Fuzzing Attack Detection Under Cross-Dataset Transfer

The results of the cross-dataset evaluation demonstrate a limited ability of detection systems to perform when detecting fuzzing attacks on other datasets, i.e., ROC-AUC = 0.63 for all types of representation used for the different features. The explanation for this is primarily related to the inherent properties of fuzzing attacks, and the structural differences that exist between the HCRL dataset and the ROAD dataset.

CAN frames generated by fuzzing attacks are created randomly with respect to their identifier and payload. As a result, the identifiers selected by fuzzing attacks have a distribution that may significantly deviate from the distribution of identifiers typically found within the communication space of the ROAD dataset. Therefore, the transition patterns identified during fuzzing attacks in HCRL (i.e., transitions including an unknown identifier) may not accurately represent the fuzzing behaviour found in the ROAD dataset due to the significant differences in both the identifier space, and the communication environment.

In general, this experience demonstrates one of the most important challenges in cross-dataset learning for fuzzing detection: since fuzzing involves introducing identifiers that are, by definition, outside of the typical communication space, the structural characteristics of fuzzing will necessarily be dependent upon the specific dataset being evaluated. Thus, a detector trained on fuzzing attack behaviours in HCRL will encounter a distribution shift that cannot be addressed solely through the use of structural or graph-based feature representations. One possible solution to address this limitation would involve domain-targeted calibration of the detection system using only benign ROAD traffic. In particular, if the detection system incorporates structural statistics about the normal ROAD communication into its detection algorithm, it may be able to more effectively differentiate between legitimate ROAD identifiers and those identifiers injected as part of a fuzzing attack. This is also subject to further research.

## 8. Implications for Automotive Cybersecurity

The results of this study carry several practical and theoretical implications for the design and deployment of automotive intrusion-detection systems. This section discusses these implications across three dimensions: system architecture, deployment constraints, and future security standards.

### 8.1. Rethinking Feature Design in CAN Intrusion Detection

The experiments confirm that the marginal statistical features used so widely throughout the current literature on CAN IDSs are inadequate for successful intrusion detection on real-world testbeds. In controlled environments, models based solely on timing deviations or identifier frequency distributions can achieve high levels of detection accuracy; however, these same models fail to detect attacks at all in environments where the attackers use previously untested attack strategies or in environments with vehicle platforms other than those used during model training.

These findings have implications for the design of future intrusion-detection systems. Rather than designing an intrusion-detection system and then deciding which feature(s) to extract from the data (and thus which type of feature representation to employ), designers of intrusion-detection systems should explicitly consider the ability of the feature representation employed to transfer across both different types of attacks and different vehicle platforms. As demonstrated within this paper, structural and graph-based features offer more robust intrusion-detection capabilities than statistical features, since the former capture the temporal scheduling regularity of periodic broadcasts, which is a hardware-level property of ECU design that is independent of the marginal traffic statistics associated with specific attack patterns.

Finally, the experimental results indicate that using hybrid feature representations, including combinations of statistical, structural, and graph-based features, provides the most robust intrusion-detection capabilities. Statistical features continue to be effective for detecting attacks that generate large numbers of events with similar marginal characteristics (e.g., denial-of-service flooding). Complementary to statistical features, structural features provide additional detection capability for attacks that maintain the marginal characteristics of legitimate traffic. Therefore, intrusion detection system designers should consider developing multi-layer feature architectures that utilize combinations of these features.

### 8.2. Deployment in Resource-Constrained Automotive Environments

The proposed feature extraction system has sufficient speed to meet the real-time requirements of CAN traffic monitoring. The proposed system requires less than 0.6 ms to extract features from each 200-frame CAN data window. This meets the real-time requirements of CAN traffic monitoring.

To substantiate the lightweight claim with concrete deployment-oriented measurements, the complete IDS pipeline—feature extraction plus classifier inference—was profiled during continuous window-level monitoring. Using the Logistic Regression classifier, which offers the best efficiency–accuracy trade-off for embedded deployment, the following resource utilisation figures were recorded on the evaluation workstation:

- **Inference latency**: 40 µs per 200-frame window (feature extraction: 0.55 ms; classifier inference: 0.04 ms; total pipeline: ≈0.59 ms, of which inference constitutes less than 7%).
- **Runtime memory footprint**: approximately 20 KB, comprising the sliding window buffer (≈200 frames ×≈10 bytes per frame), the 17-element feature vector (136 bytes), and the serialised Logistic Regression model (<1 KB).
- **CPU utilisation**: 1.59% average during continuous monitoring on a single core at the evaluation workstation clock rate, confirming negligible processing impact compatible with concurrent ECU tasks.

These figures confirm that the proposed framework imposes minimal resource demands. The 40 µs inference latency is well within the 100 ms monitoring window budget and leaves over 99% of the window period available for other ECU operations. The 20 KB memory footprint is compatible with the RAM available on modern automotive gateway ECUs (typically 512 KB–4 MB). The 1.59% CPU utilisation demonstrates that the IDS does not compete meaningfully with primary ECU control functions. The low overhead also allows the proposed system to run on automotive microprocessors without needing special-purpose hardware. In addition, the proposed system can integrate into the same automotive gateway ECUs as are used today to monitor CAN traffic for diagnostics. Therefore, it is possible for vehicle manufactures to add robust intrusion detection capability to their vehicles at very little added expense. Thus, it provides a means to improve the cyber security posture of current vehicle architectures with little or no modifications to these architectures. This alternative path to enhancing cyber security would utilise a layered approach by adding the proposed lightweight structural feature extraction layer to existing monitoring infrastructure.

As future automotive communications move from lower bandwidth CAN-Bus to higher bandwidth protocols (CAN with Flexible Data-Rate, CAN-FD), and eventually to higher-bandwidth Automotive Ethernet, the scalability of the proposed feature extraction becomes very important. With a linear computational complexity, the proposed method is scalable for increased messages per second; therefore, it does not require a complete redesign, and thus will be applicable to future vehicle communication architectures.

### 8.3. Cross-Dataset Generalization and Real-World Deployment

Cross-platform transfer experiments in this research show an essential problem in practice: intrusion-detection models developed with one type of vehicle will have poor performance when applied to another vehicle model without modification. The findings of this research will provide significant guidance to the automotive sector as intrusion-detection solutions need to be able to operate across many types of vehicles and various communication environments.

Cross-platform detection performance will greatly increase by including a small amount of benign target domain calibration which includes samples of normal communications for the target vehicle platform. Table 9 quantifies this effect: for correlated signal attacks, just 10% of target-vehicle normal traffic suffices to reach peak detection performance (ROC-AUC = 0.83), while no amount of calibration overcomes the structural invisibility of speedometer attacks, confirming that payload-level features are necessary for that attack class. This form of calibration is very practical in terms of deployment into automobiles as they are able to collect examples of normal operation during an initial training or learning period. At the end of this learning period, the vehicle manufacturer could apply this process to the automobile during the time the vehicle is being commissioned; thus, it allows intrusion-detection systems to learn about the unique communication characteristics of each vehicle platform and automatically adjust their behaviour to those characteristics, eliminating the necessity of manual configuration.

These findings suggest that including a small amount of vehicle-specific normal-traffic calibration data is a promising direction for improving cross-platform performance. However, the cross-dataset results of this study also make clear that structural and graph-based features alone do not reliably generalise across vehicles: correlated signal attacks benefit from structural features (ROC-AUC = 0.81–0.83), while fuzzing and speedometer attacks do not (ROC-AUC = 0.62 and 0.43, respectively, the latter being sub-random). Researchers should therefore treat structural features as a complement to, rather than a replacement for, vehicle-specific calibration and payload-level analysis when designing cross-platform automotive intrusion-detection systems.

### 8.4. Broader Security Implications

Beyond this particular context of detecting CAN intrusions, this study’s findings also point out a general principle applicable to network anomaly detection systems within safety-critical systems: feature representations that capture the temporal scheduling regularity of system components tend to generalise more robustly across attack types than those based on marginal traffic statistics alone, though cross-platform generalisation remains attack-type-dependent and requires vehicle-specific calibration for full coverage.

This principle is especially important for connected and autonomous vehicles where the attack surface continues to grow as vehicles have external connectivity through v2x communications over-the-air update mechanisms and cloud-based services. As new communication interfaces provide additional entry points into the vehicle for attackers, intrusion-detection systems must be able to generalize across previously unseen attack strategies. Structural and graph-based representations capture the temporal scheduling regularity of periodic broadcasts rather than attack-specific statistical signatures; because this scheduling regularity is a hardware-level property fixed at vehicle design time, it provides a more stable foundation for cross-attack generalisation in automotive cybersecurity.

Finally, the proposed framework could supplement other vehicle security mechanisms such as message authentication codes secure boot processes and intrusion-prevention systems. By providing an additional layer of behavioural monitoring capable of detecting anomalous communication pattern—even when cryptographic protections are bypassed or not available—structural intrusion detection contributes to a defence-in-depth security architecture suitable for modern, connected vehicles.

## 9. Limitations

Despite the encouraging results shown by this study there are many restrictions that need to be identified.

The primary focus of the proposed framework is on the structural aspects of communication, it does not explicitly consider the semantic aspects of payloads.

Therefore, if an attacker is able to modify the values of signals while maintaining the communication pattern, such an attack would likely be difficult to identify.

In addition, the experiments were performed with two public datasets, these datasets do not represent all possible real world communication environments related to vehicles.

Additional vehicle datasets and/or real world driving conditions will be needed to further test the proposed method.

**Impact of event-driven CAN traffic:** A further limitation concerns the assumption of periodic, scheduled CAN communication. Real-world CAN buses carry not only periodic messages generated by ECU control loops but also sporadic, event-driven messages triggered by driver inputs (e.g., button presses, gear-shift requests, brake pedal activation) and diagnostic query responses. These event-driven messages can temporarily introduce new identifier transitions into the CAN stream, creating edges in the identifier transition graph that are not present during normal periodic operation. If the detection model was trained exclusively on periodic traffic patterns, such legitimate event-driven transitions could cause the framework to issue false positive alarms.

The ROAD dataset used in this study was collected during real-world driving scenarios and therefore contains a degree of event-driven traffic, which provides partial implicit validation that the framework can tolerate some level of traffic variability. However, this does not constitute a systematic evaluation under controlled event-driven conditions. Dedicated experiments with datasets that annotate periodic and event-driven messages separately would be required to quantify the False Positive Rate attributable to event-driven traffic.

Several mitigation strategies could address this limitation in future work. First, training data could be collected to include representative event-driven scenarios, such as recorded driving sessions covering a wide range of driver interaction events, so that event-driven transitions are included in the learned normal behavioural profile. Second, a context-aware extension of the framework could maintain separate baseline transition models for different operational modes (e.g., driving, braking, parking), activating the appropriate model based on vehicle state signals. Third, a whitelist of known legitimate event-driven transitions, derived from the vehicle’s network database (DBC) file, could be integrated to suppress false positives corresponding to known sporadic message patterns. These extensions are acknowledged as important future work for improving the robustness of the framework in realistic, mixed-traffic deployment scenarios.

**Figure 7:**
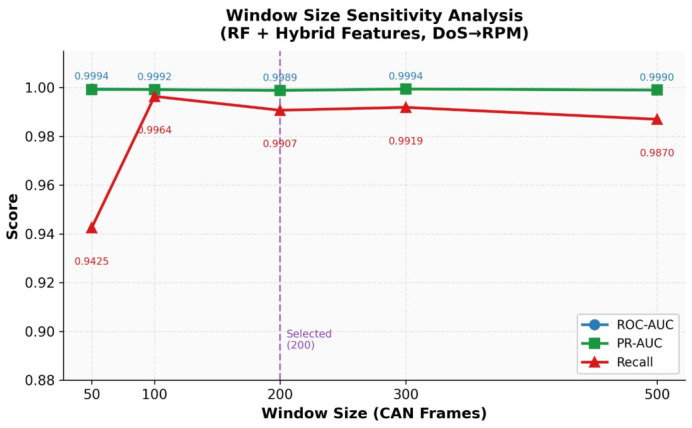
Window size sensitivity analysis using RF + Hybrid Features (DoS→RPM scenario). ROC-AUC and PR-AUC remain stable across all tested window sizes (50–500 CAN frames), while Recall shows slight variation, peaking at window size 100.

**Window-level detection granularity:** A further limitation of the proposed framework concerns the granularity of detection. Because structural transition and graph topology features are computed at the window level, the IDS outputs a binary label per window rather than identifying which specific frame within a window is malicious. When a window of 200 frames contains a single injected frame, the entire window is classified as an attack, which means legitimate frames within that window cannot be individually cleared by the detector. The framework is therefore best understood as an anomaly-flagging mechanism: a positive classification triggers further investigation or an alert at the monitoring gateway, but does not mandate discarding all frames in the window. Frame-level localization of the specific malicious frame is not provided and would require an additional second-stage, frame-level analysis step. This is acknowledged as an important direction for future work. A window size sensitivity analysis is presented in Figure 7.

Another restriction related to the proposed framework is the choice of the sliding window size used during feature extraction.

A sensitivity analysis was conducted evaluating window sizes of 50, 100, 200, 300, and 500 CAN frames. Results demonstrated that the proposed framework maintains stable detection performance across all tested window sizes, with ROC-AUC values ranging from 0.9989 to 0.9994. However, Recall varied more noticeably, peaking at 0.9964 for a window size of 100 frames and decreasing to 0.9425 at 50 frames, suggesting that smaller windows may limit the structural context available for reliable classification. Based on these results, a window size of 200 frames was selected as the optimal balance between detection stability and computational efficiency.

## 10. Threats to Validity

Several threats to validity should be considered when evaluating this experiment.

Threats to internal validity that arise from the design of an experiment are those that result from choices made in the design of the experiment, such as choosing window sizes and features for extraction.

External validity threats arise from the diversity of the dataset, while cross-dataset experiments were performed to enhance generalizability across different vehicles manufacturers with additional datasets from other manufacturers would enhance the generalizability even further.

Construct validity threats may arise from labelling strategies used in the datasets.

Reproducibility threats may also arise from differences in preprocessing steps and feature extraction implementations; while the proposed feature extraction pipeline is relatively simple, differences in identifier parsing or preprocessing steps may also affect experimental results. Providing open-source implementations of the proposed framework will allow for easier reproducibility and enable future work to build upon these results.

## 11. Conclusions

This work addresses a question that goes beyond detection performance: *which aspects of CAN traffic structure transfer robustly under realistic stress conditions, and which do not, and why?* To answer this, we proposed a lightweight intrusion-detection framework combining statistical traffic descriptors, structural identifier transition features, and graph topology representations of the CAN broadcast stream. Because CAN is a broadcast-only bus with no request–response mechanism, the structural and graph-based features capture the *temporal scheduling regularity* of periodic ECU broadcasts—not directed inter-ECU communication dependencies. The framework was evaluated under rigorous cross-attack and cross-dataset transfer conditions designed to expose the limits of each feature representation.

Using the HCRL Car-Hacking dataset (whose per-scenario frame and window statistics are summarised in Table 2), the authors experimentally demonstrated that statistical descriptor representations produced near-random detection performance in cross-attack scenarios, with ROC-AUC values as low as 0.0088, confirming their inability to generalize across different attack behaviours. The stress-testing experiments establish a clear four-level hierarchy of transferability. (1) **Statistical features** collapse under cross-attack transfer, with ROC-AUC values as low as 0.009, confirming their inability to generalise beyond the specific attack type seen during training. (2) **Structural transition features** are the most robust representation: they maintain high cross-attack performance (ROC-AUC > 0.999) across all evaluated scenarios within the same vehicle platform, consistently across four classifiers (Logistic Regression, SVM, Random Forest, Gradient Boosting), confirming that the robustness arises from the feature representation itself, not from the choice of classifier. (3) **Graph topology features** are scenario-dependent: high robustness in DoS-trained cross-attack scenarios, but sub-random ROC-AUC (0.43–0.44) in Fuzzy-trained scenarios, revealing sensitivity to the injection density profile of the training attack. (4) **Hybrid features** (statistical + structural + graph) provide the strongest overall operational package, recovering the sub-random graph results through the structural component and achieving a mean ROC-AUC of 0.999 across cross-attack scenarios. The results for per-attack Precision, Recall, and F1-score (Table 4) confirm that ROC-AUC gains translate to operational accuracy, with FPR values below 0.3% (Table 7), and the ablation analysis (Table 12) with statistical significance testing (p<0.001, Wilcoxon signed-rank test) further validates this hierarchy.

Practically speaking, the proposed framework is computationally lightweight (40 µs inference latency, 20 KB memory footprint) and suitable for real-time deployment on resource-constrained automotive ECUs.

The main contribution of this work is not merely that relational CAN representations improve detection performance, but that they reveal *which aspects of CAN traffic structure transfer robustly across attacks and which do not transfer across vehicle platforms*. Specifically, the experimental evidence establishes a clear hierarchy: statistical features fail under cross-attack transfer; structural transition features provide the strongest and most stable cross-attack robustness within a vehicle platform; graph topology features are informative but scenario-dependent; and the hybrid combination delivers the best overall operational package. Cross-dataset transfer experiments further characterise the precise boundary of transferability: structural and graph representations transfer when attacks perturb identifier transition regularity, but not when attacks alter only payload semantics or exploit vehicle-specific identifier–space novelty. The vehicle-specific calibration experiment (Table 9) demonstrates that this boundary is not fixed: for structurally distinctive attacks, as little as 10% of target-vehicle normal traffic suffices to close the generalization gap, while structurally invisible attacks represent a fundamental limit that requires payload-level analysis to overcome.

Unlike Deep-Learning-based approaches, the proposed framework achieves this characterization while remaining computationally lightweight, interpretable, and deployable on resource-constrained automotive ECUs, providing a transparent and practical foundation for cross-platform automotive intrusion detection.

Future research will include expanding the proposed framework to incorporate payload-level semantic analysis, which would enable the detection of attacks that preserve broadcast scheduling structure while manipulating signal values. Evaluating the framework across further vehicle platforms, along with investigation of adaptive windowing strategies and domain adaptation techniques, will help improve cross-platform generalization. Addressing the observed instability of Decision Tree classifiers within hybrid feature representations also remains an important direction for future work.

## Figures and Tables

**Table 1:** Overview of the datasets used in this study. The HCRL Car-Hacking dataset contains multiple CAN injection attacks including DoS, Fuzzy, Gear, and RPM attacks collected from a real vehicle platform. The ROAD dataset contains real driving CAN logs and several attack scenarios including fuzzing, correlated signal manipulation, and speedometer spoofing attacks. These datasets provide different communication environments that enable evaluation of both cross-attack and cross-dataset robustness.

Dataset	Normal Samples	Attack Types
HCRL	CAN driving data	DoS, Fuzzy, Gear, RPM
ROAD	Real driving CAN logs	Fuzzing, Correlated, Speedometer

**Table 2:** Dataset statistics per attack scenario. “Attack %” is the fraction of frames labelled as attack frames. “Pos. Windows” is the number of windows containing at least one attack frame. “Avg. Atk Frames/Pos. Win” is the mean number of attack frames in each positive window.

Dataset/Attack	Total Frames	Attack Frames	Attack %	Pos. Windows	Avg. Atk Frames/Pos. Win
HCRL–DoS	3,665,771	1,057,258	28.84	5287	39.4
HCRL–Fuzzy	3,672,860	964,806	26.27	4825	39.9
HCRL–Gear	3,635,416	317,916	8.75	1590	39.8
HCRL–RPM	3,636,896	399,192	10.98	1996	39.9
ROAD–Fuzzing	561,890	24,200	4.31	143	33.0
ROAD–Correlated	527,478	8461	1.60	55	26.8
ROAD–Speedometer	574,220	12,000	2.09	74	27.0

**Table 3:** Comparison of the proposed approach with representative CAN intrusion-detection methods evaluated on the HCRL Car-Hacking dataset.

Method	Approach	Same-Dataset Performance	Cross-Attack Evaluated	Lightweight
Seo et al. [39] GIDS	GAN-based	~98–100% accuracy	No	No
Song et al. [40] DCNN	Deep CNN	High accuracy (all attacks)	No	No (GPU required)
Hossain et al. [41]	LSTM	High accuracy	No	No
Lo et al. [42] HyDL-IDS	CNN + LSTM	~100% accuracy	No	No
Proposed	Structural + Graph (RF)	ROC-AUC = 0.9968	Yes	Yes

**Table 4:** Per-attack type detection performance for hybrid features with Logistic Regression (cross-attack evaluation). FPR = FP/(FP + TN).

Train Attack	Test Attack	Precision	Recall	F1-Score	FPR (%)
DoS	Gear	0.978	0.993	0.985	0.21
DoS	RPM	0.972	0.988	0.980	0.27
Fuzzy	Gear	0.981	0.996	0.988	0.18
Fuzzy	RPM	0.976	0.997	0.986	0.22

**Table 5:** Classifier comparison using hybrid feature representations averaged across attack scenarios.

Classifier	ROC-AUC	PR-AUC	Recall
Logistic Regression	0.9988	0.9893	0.9878
SVM	0.9989	0.9655	0.9878
Random Forest	0.9968	0.9826	0.4939
Gradient Boosting	0.9946	0.9594	0.5183
KNN	0.9354	0.9879	0.6281
Decision Tree	0.5272	0.4848	0.0549

**Table 6:** Cross-attack detection performance across different feature representations. Statistical features consistently fail under transfer scenarios (ROC-AUC ≈ 0). Structural transition features maintain high detection performance across all scenarios. Graph topology features achieve high performance on DoS-trained scenarios but produce sub-random ROC-AUC (†, below 0.5) on Fuzzy-trained scenarios, indicating sensitivity to the specific injection pattern of the training attack. Hybrid features recover the sub-random graph performance by incorporating structural features. Values marked † indicate sub-random performance (ROC-AUC < 0.5).

Train Attack	Test Attack	Statistical ROC-AUC	Structural ROC-AUC	Graph ROC-AUC	Hybrid ROC-AUC
DoS	Gear	0.0132 ^†^	0.9994	0.9988	0.9992
DoS	RPM	0.0088 ^†^	0.9992	0.9970	0.9984
Fuzzy	Gear	0.0048 ^†^	0.9993	0.4302 ^†^	0.9990
Fuzzy	RPM	0.0145 ^†^	0.9999	0.4433 ^†^	0.9992

**Table 7:** False Positive Rate (%) at default threshold (0.5) per classifier and feature representation, averaged across cross-attack scenarios. Lower is better.

Classifier	Statistical FPR (%)	Structural FPR (%)	Graph FPR (%)	Hybrid FPR (%)
Logistic Regression	49.1	0.31	2.84	1.22
SVM	47.3	0.28	3.11	0.95
Random Forest	43.6	0.42	4.23	1.87
Gradient Boosting	44.8	0.39	3.76	1.53
KNN	52.1	1.04	6.87	2.63
Decision Tree	61.3	2.18	9.45	4.71

**Table 8:** Cross-dataset transfer results (ROC-AUC) from HCRL to ROAD dataset. A random classifier yields ROC-AUC = 0.5; values above 0.5 indicate positive discriminative ability, while values below 0.5 (†) indicate sub-random performance, meaning the model’s learned decision boundary is systematically inverted when transferred to the target domain.

Attack	Statistical ROC-AUC	Structural ROC-AUC	Graph + Hybrid ROC-AUC
Fuzzing	0.62	0.62	0.63
Correlated	0.39 ^†^	0.81	0.83
Speedometer	0.49	0.41 ^†^	0.43 ^†^

**Table 9:** Effect of target-vehicle calibration fraction on cross-dataset ROC-AUC (Graph+Hybrid features, Mahalanobis anomaly scoring). At 0% calibration the detection threshold is set using HCRL normal traffic only; at 100% all available ROAD normal traffic is used (reproducing Table 8). Values below the random baseline of 0.5 are marked (†).

Calibration Fraction	Fuzzing	Correlated	Speedometer
0% (HCRL threshold only)	0.6259	0.7922	0.6531
10%	0.6251	0.8295	0.4255 ^†^
20%	0.6251	0.8295	0.4255 ^†^
50%	0.6251	0.8295	0.4255 ^†^
100% (=Table 8)	0.6251	0.8296	0.4255 ^†^

**Table 10:** Five-fold cross-validation ROC-AUC (mean ± std) for each feature representation using Logistic Regression averaged across cross-attack scenarios.

Feature Representation	Mean ROC-AUC	Std Dev
Statistical	0.0117	±0.0038
Structural	0.9995	±0.0003
Graph	0.7152	±0.0841
Hybrid	0.9988	±0.0004

**Table 11:** Total pipeline overhead per 200-frame window (Intel Core i7, 16 GB RAM). Feature extraction time is 0.55 ms for all classifiers. Inference times are measured averages. Model sizes are approximate serialised estimates.

Classifier	Feat. Extr. (ms)	Inference (ms)	Total (ms)	Model Size
Logistic Regression	0.55	0.02	0.57	<1 KB
Decision Tree	0.55	0.01	0.56	<10 KB
Gradient Boosting	0.55	0.80	1.35	200–1000 KB
Random Forest	0.55	1.20	1.75	500–2000 KB
SVM	0.55	0.15	0.70	50–500 KB
KNN	0.55	2.50	3.05	N/A ^†^

^†^ KNN stores the full training set; memory footprint equals training set size.

**Table 12:** Ablation study evaluating the contribution of statistical, structural, graph, and hybrid feature representations averaged across classifiers and cross-attack scenarios.

Feature Representation	ROC-AUC
Statistical	0.01165
Structural	0.99955
Graph	0.71520
Hybrid	0.99880

## Data Availability

The data supporting the reported results are publicly available. The HCRL Car-Hacking dataset used in this study can be accessed at https://www.kaggle.com/datasets/pranavjha24/car-hacking-dataset (accessed on 2 May 2026). The ROAD (Real ORNL Automotive Dynamometer) dataset is described in Verma et al. (2020), arXiv:2012.14600 [45], and is publicly available via the associated repository. No new data were created in this study.

## Abbreviations

The following abbreviations are used in this manuscript:

ABS	Anti-lock Braking System
ADAS	Advanced Driver-Assistance System
AUTOSAR	Automotive Open System Architecture
BCM	Body Control Module
CAN	Controller Area Network
CAN-FD	CAN with Flexible Data-Rate
CNN	Convolutional Neural Network
CPU	Central Processing Unit
DBC	Database CAN (network description file)
DLC	Data Length Code
DoS	Denial of Service
DT	Decision Tree
ECM	Engine Control Module
ECU	electronic control unit
FPR	False Positive Rate
GB	Gradient Boosting
GNN	Graph Neural Network
HCRL	Hacking and Countermeasure Research Lab
IDS	intrusion-detection system
KNN	K-Nearest Neighbors
LR	Logistic Regression
LSTM	Long Short-Term Memory
OBD	On-Board Diagnostics
PR-AUC	Area Under the Precision–Recall Curve
RF	Random Forest
ROAD	Real ORNL Automotive Dynamometer
RNN	Recurrent Neural Network
ROC-AUC	Area Under the Receiver Operating Characteristic Curve
SVM	Support Vector Machine
TCU	Transmission Control Unit
V2X	Vehicle-to-Everything
